# Eysenck's Two Big Personality Factors and Their Relationship to Depression in Patients with Chronic Idiopathic Pain Disorder: A Clinimetric Validation Analysis

**DOI:** 10.5402/2012/140458

**Published:** 2012-09-04

**Authors:** Per Bech, Marianne Lunde, Stine Bjerrum Møller

**Affiliations:** Psychiatric Research Unit, Mental Health Centre North Zealand, University of Copenhagen, 3400 Hillerød, Denmark

## Abstract

*Aim*. The clinimetric aspects of Eysenck's two big personality factors (neuroticism and extraversion) were originally identified by principal component analysis but have been insufficiently analysed with item response theory models. Their relationship to states of melancholia and anxiety was subsequently analysed. *Method.* Patients with chronic idiopathic pain disorder were included in the study. The nonparametric item response model (Mokken) was compared to the coefficient alpha to validate the anxiety and depression subscales within the neuroticism scale and the extraversion and introversion subscales within the extraversion scale. When measuring states of depression and anxiety, the Melancholia Scale and the Hamilton Anxiety Scale were used. *Results*. We identified acceptable subscales of anxiety and depression in the Eysenck factor of neuroticism and extraversion versus introversion subscales within the Eysenck factor of extraversion. Focusing on the item of “Does your mood often go up and down?” we showed a statistically significant association with melancholia and anxiety for patients with a positive score on this item. *Conclusion*. Within the Eysenck factor of neuroticism it is important to differentiate between the anxiety and depression subscales. The clinimetric analysis of the Eysenck factor of extraversion identified valid subscales.

## 1. Introduction

Clinical psychometrics is clinimetrics in psychiatry [1]. The term *clinimetrics* was introduced by Feinstein [2] to cover the clinical markers in clinical medicine which cannot be measured by a biophysicist, a geneticist, or a pharmacologist. From a clinical point of view such markers as neuroticism or depression have high validity. Using experienced psychiatrists [3] as an index of validity, Eysenck's neuroticism proved to have a higher validity than many of the personality dimensions covered by Murray's theory [4]. We also found that a subgroup of the items in the Hamilton Depression Scale was used by experienced psychiatrists when assessing depression severity; this led to the Melancholia Scale (MES) [1, 5]. 

The psychometric validation procedure which employs mathematical or statistical models to evaluate the measurement aspects of questionnaires such as the Eysenck Personality Questionnaire (EPQ) [6] or the Melancholia Scale (MES) [7] typically includes factor analysis, Cronbach's coefficient alpha, or item response theory models [1]. 

When identifying the two-factor structure of personality, namely, neuroticism and extraversion, Eysenck used principal component analysis [8]. At their release of the EPQ, Eysenck and Eysenck [6] had difficulties with the use of the alpha coefficients: “It is always possible to achieve very high alpha coefficients by simply using questions which are merely variants of one single and very restricted theme…”

The psychometric problem of using positively and negatively phrased items was investigated by Eysenck and Eysenck [8], resulting in the conclusion that when measuring neuroticism it is most valid to use the negatively phrased items. Therefore, the 23 items in the EPQ Neuroticism scale are all to be considered as symptoms. In the 21-item Extraversion scale three items are inverted to cover introversion.

In the present study, in which we focus on clinimetrics, we have selected the nine items covering anxiety in the EPQ Neuroticism scale and the remaining fourteen items covering depression [1]. These two neuroticism subscales are shown in Table 1. As regards the Extraversion scale in EPQ we have selected the nine items which we considered to be clinically valid in covering the entire dimension of extraversion (E_9_); taking into account the local dependency between items [1]. The remaining nine items (R_9_) in Table 2 seem merely to be variants on the theme of extraversion already covered by our selected nine items (Table 2). The three inverted items of the EPQ extraversion scale can be considered as a scale measuring introversion (I_3_, Table 2).

In a study on patients with chronic idiopathic nonmalignant pain disorder fulfilling the DSM-III criteria for this disorder, we have used both the original EPQ questionnaire and the Melancholia Scale (MES) [9–12]. In our original study [9] we included patients with chronic idiopathic pain disorder to test the hypothesis that these patients might have chronic idiopathic pain as a variant of depressive illness. However we showed [9] that only 10% of the included patients actually had a clinical depression. On the other hand, we realized [12] that approximately 50% of the patients had clinical anxiety. On this background we considered these patients as an appropriate population when studying the Eysenck factor of neuroticism and extraversion in relation to depression or anxiety. In the present analysis we have evaluated the psychometric validity of the neuroticism anxiety subscale, the neuroticism depression subscale, and the three subscales in the extraversion/introversion domain (Table 2). We evaluate the predictive validity of these scales in the identification of depression as measured by the Melancholia Scale (MES) and the Hamilton Anxiety Scale (HAM-A) [1]. 

## 2. Materials and Methods

### 2.1. Patients

The study has been described in detail elsewhere [9, 11, 12]. In brief, the protocol inclusion criterion was chronic idiopathic nonmalignant pain fulfilling the DSM-III criteria for the diagnosis of chronic idiopathic disorder. The exclusion criteria included severe hypertension, cardiovascular disorders, liver or kidney disorders, micturition disturbances, glaucoma, and anticoagulant therapy. The psychiatric exclusion criteria were organic brain disorders, alcohol or other substance use disorders, schizophrenia, and bipolar affective disorders as well as primary or endogenous depression as defined by a Newcastle Depression Scale [13] diagnostic depression score of 6 or more.

The baseline characteristics as to the types of pains, and of age, and gender in the Loldrup et al. [9] study with a total of 253 patients are shown in Table 3.

### 2.2. Clinical Assessment Scales

In the present study on the relationship between the Eysenck personality dimensions of neuroticism and extra-/introversion, we have focused on symptom scales (the Hamilton Anxiety Scale (HAM-A), the Melancholia Scale (MES), and the specific depression items in the Beck Depression Inventory (BDI_6_) all these scales in the versions released by Bech [1]) besides the corresponding items from the EPQ [6].

### 2.3. Observer (Interview-Based) Rating Scales

#### 2.3.1. The Melancholia Scale (MES)

The Bech-Rafaelsen Melancholia Scale ([1, 7] contains eleven items and has a window (time frame) of the past 3 days. The scale is derived from the Hamilton Depression Scale and covers the specific depression items corresponding to the symptoms included in DSM-IV major depression. The scale fulfils the item response theory model [14]; that is, the total score is a sufficient statistic [1]. With less than 10% of the patients in the Loldrup et al. [9] study fulfilling criteria for major depression using the MES and a mean score of 7.2 with a standard deviation of 4.4, the sample has a subclinical level of depressive symptoms.

#### 2.3.2. The Hamilton Anxiety Scale (HAM-A)

We have used the version accepted by Hamilton [1]. The HAM-A contains 14 items with a window (time frame) of the past 3 days. With a mean of 11.3 and a standard deviation of 5.9, the sample has higher anxiety scores than the general population [12].

Furthermore, we have focussed on the subscale within the HAM-A, namely, the psychic anxiety scale, however without the item of depressed mood and the item of sleep (HAM-A_6_) [1].

### 2.4. Patient-Administered Questionnaires

#### 2.4.1. Symptom Scale

We have focused on the six items covering the most specific items in the Beck Depression Inventory (BDI_6_) [1, 12, 15]. The BDI_6_ items correspond to the HAM-D_6_ [1]. The window (time frame) of the BDI is “here and now,” that is, the status on the test day [15].

### 2.5. Personality Scales

We have used the Eysenck Personality Questionnaire (EPQ) as published by Eysenck and Eysenck [6]. The sample has a significantly higher neuroticism score than the general population, as accounted for elsewhere [12]. The 23 neuroticism items are shown in Table 1, divided into anxiety-related items and depression-related items. The 21 extraversion/introversion items are shown in Table 2; here the nine specific extraversion items (E_9_), the three introversion items (I_3_), and the remaining extraversion items (R) are indicated. The window (time frame) of the EPQ is “life time,” that is, to disregard the “here and now” situation and to consider the habitual trait behaviour.

### 2.6. Psychometric Validation Analysis

Principal component analysis (PCA) was performed in an attempt to collect all useful common variance in the EPQ neuroticism and extraversion subscales. As regards the neuroticism subscale, it was expected that the first principal component would be a general factor because all the items (N_23_) were selected as having positive intercorrelation. If the second or the third component still had an eigenvalue above 1, it was expected that the second or the third factor would be bidirectional. This part of the analysis tested to what extent the anxiety items and the depression items (Table 1) were separated in the PCA by different factor loadings, that is, negative versus positive loadings. If this was the case, no rotation procedure was found necessary [1, 16, 17].

In the extraversion scale (Table 2), it was expected that the three introversion items would have loadings with signs different from those of the extraversion items within the first principal component. If this was the case, it would in itself be a test of compliance as to correct completion of the questionnaire [1]. 

Cronbach's coefficient alpha [6, 18] was used to test the hypothesis that the full Extraversion scale contains too many items that are merely variants of one single section of the dimension of extraversion [1].

The Mokken analysis was used to test to what extent the total summed item score was a sufficient statistic. This is a nonparametric item response theory model based on Loevinger's coefficient of homogeneity [19, 20]. According to the Mokken analysis [21, 22], the resulting coefficient of homogeneity is calculated as the weighted average of the individual coefficients [7, 23]. Mokken recommended that a coefficient of homogeneity from 0.30 to 0.39 should be regarded as just acceptable, while a coefficient of 0.40 or higher should clearly be interpreted as a demonstration of unidimensionality; that is, that the items are additive and their summed total score a sufficient statistic. 

## 3. Results

In the original study, 253 patients fulfilled the inclusion and exclusion criteria, but in this analysis we have solely focused on the patients without missing item scores in any of the rating scales or questionnaires under examination. We had missing answers for 14 patients (or 5.5%); thus our results are based on 239 patients. Comparing the included 239 patients with the 14 patients with missing items revealed no statistically significant difference in depression (MES mean 8.3) and no difference in years of age (mean 49.3 versus 50.2) or gender (*P* = 0.918).

Table 1 shows the mean score on the 23 items from Eysenck's neuroticism scale. The items are listed in Table 1 when ordered in decreasing prevalence. The most inclusive individual item (highest prevalence) is No. 84 (bubbling energy/very sluggish). The principal component analysis identified six components with an eigenvalue of 1 or higher. The first principal component obtained an eigenvalue of 6.83, the second 1.76, and the third 1.32. Together these three components explained 43.1% of the variance. Table 1 shows the item loadings of the first principal component. They are all positive, indicating that we have a general factor of neuroticism. The anxiety items tend to have higher prevalence than the depression items (higher mean score).

Table 4 shows the third principal component with an eigenvalue of 1.32. (the second principal component was clinically less clear than the third). The items with negative loadings in Table 4 cover the depression aspect of neuroticism (with 8 depression items and 2 anxiety items). The positively loaded items in Table 4 cover the anxiety-related items in the Neuroticism scale (although the prevalence of these items is rather mixed, with 7 anxiety items and 6 depression items).

Table 2 shows the principal component analysis of the Extraversion scale. As expected, the three items covering introversion were negatively loaded on the first principal component which has an eigenvalue of 5.60. The principal component analysis identified six components with an eigenvalue of 1 or higher. Neither the second nor the third component gave acceptable clinical meaning.

Table 5(a) shows the Cronbach coefficient alpha and the coefficient of homogeneity of the two subscales from the Extraversion scale. The full 21 items had a high alpha but a very low coefficient of homogeneity. Both the E_9_ and the I_3_ subscale have an adequate coefficient of homogeneity.

Table 5(b) shows the coefficient of homogeneity of the two Neuroticism subscales as well as the total 23-item scale. The depression subscale N_14_ has a coefficient of homogeneity comparable with the extraversion subscales in Table 4(a). Thus, the N_23_ had the highest alpha coefficient (0.88), but the coefficient of homogeneity was lower than that obtained by N_14_.

Table 6 shows the intercorrelations between the subscales from the Neuroticism and Extraversion scales with the depression scales (MES/BDI_6_) and anxiety scale (HAM-A). A clear and significant difference was seen between extraversion (E_9_) and introversion (I_3_). A tendency to higher coefficients was seen for the depression subscale of neuroticism (N_14_) compared to the anxiety subscale of neuroticism (N_9_) for the two depression scales (MES and BDI_6_), with an opposite finding for N_9 _(anxiety) and HAM-A. 

In his item responses analysis of the first version of the neuroticism scale [25, 26], Andrich [26] identified item 3 in Table 1 as a relatively less prevalent item than item 84 in Table 1, which is in accordance with our results (mean scores in Table 1). When using item 3 as indicator (Table 7), we showed a significant relationship between a positive response to item 3 and depression (MES) and both HAM-A_6_ and total HAM-A_14_.

## 4. Discussion

In psychosomatic research Eysenck's neuroticism dimension has proved to be the most useful personality scale, resulting in attempts being made to identify Eysenck's dimension in other personality scales such as the Minnesota Multiphasic Personality Inventory (MMPI) [27, 28]. In both attempts, the anxiety-related items clearly outperformed the depression-related items in the Eysenck neuroticism concept.

In our previous study on patients with duodenal ulcer we demonstrated [28] that neuroticism was a reaction to the clinical symptoms of this disease, not an etiological factor.

In the present study on patients with chronic idiopathic pain disorder, we have demonstrated that Eysenck's final version of his neuroticism scale [6] is a rather broad construct with a nine-item subscale containing anxiety-related symptoms (N_9_) and a fourteen-item subscale with depression-related symptoms (N_14_). The coefficient of homogeneity was found more acceptable for N_14_ than for N_9_ (Table 5(b)). The correlations between the depression-related N_14_ and the depression scales (MES or BDI_6_) were higher than those obtained for the anxiety-related N_9_ (Table 6) indicating a closer affiliation between the N_14_ and depression than between N_9_ and depression. The difference was modest; however this may be explained by two circumstances: (1) that the sample had a low prevalence of depressive symptoms and a higher prevalence of anxious symptoms and (2) that depression and anxiety are highly correlated conditions [29]. 

Discrimination between anxiety and depression is often problematic because these are highly overlapping syndromes. Thus in the comprehensive study by Grinker et al. [30], anxiety was found to be a core item of depression, and in our clinical validation study of the Hamilton Depression scale [31], the item of psychic anxiety (worrying) was found to be a core item of depression. When identifying a specific depression factor in the Symptom Checklist 90 (SCL-90), the item of worry was included as a core item of depression [32].

By this psychometric refinement of the neuroticism construct, uncovering an anxiety-related subscale and a depression-related subscale, we aimed to provide a clear and unambiguous measurement of neuroticism as this will provide the opportunity for clinical validity to be improved. In future studies, the clinical validity of an anxiety-related and a depression-related neuroticism subscale should be explored in more detail. Within the research focused on constructs such as rumination, which can be characterized as pondering about the depressive symptoms, and causes and consequences of these symptoms [33] and on worry, which is characterized as an apprehensive expectation of possible negative outcome of future events [34], substantial correlations between rumination and worry have been found in clinical as well as nonclinical samples [35, 36]. Both worry and rumination have repeatedly been found to correlate significantly with anxiety as well as depression; however, factor analyses have identified two distinct constructs [37]. Some evidence suggests a stronger relationship between worry and both anxiety and depression than between rumination and depression and anxiety [38]. There is accumulating evidence that worry and rumination are linked to neuroticism [39], and in a nonclinical sample, Muris et al. [36] found support for a mediational model in which neuroticism is associated with worry and rumination which in turn are associated with anxiety and depression. Future research may focus on the relationship between the 9 anxiety-related items of the neuroticism measure that in this study demonstrated sound psychometric qualities and their relation to worry and anxiety, as well as the association between the 14 depression-related items that demonstrated excellent psychometric qualities in the present study and their relation to rumination and depression. Studies should be conducted in clinical settings and preferably utilising a longitudinal approach to test causality. 

The clinimetric analysis of the Eysenck extraversion scale showed that it is important to separate the items measuring introversion from the many extraversion items. The first principal component (Table 2) showed that the three introversion items were loaded opposite to the extraversion items, indicating a high compliance on the part of the patients. Moreover, the psychometric analysis with the coefficient of homogeneity versus Cronbach's alpha demonstrated that we have a high local dependency within the universe of extraversion items (Table 5(a)). The correlations showed the expected association, namely, that extraversion is negatively correlated with depression. As such, moderate levels of extraversion may function as inoculation against depression. On the other hand, more extreme levels of extraversion have been associated with bipolarity [40].

Regarding introversion, investigating changes in personality as a result of depressive symptoms, Shea et al. [41] found an association between number and length of depressive episodes and introversion. This indicates that the introversion construct may prove clinically useful as an index of the consequences of depressive symptoms. Bagby et al. [42] reported that low extraversion (high introversion) was associated with lack of remission. In addition, the introversion items resemble social withdrawal and avoidance behaviour, which are known correlates of depression and in the cognitive behavioural understanding of depression are conceived as factors that maintain and exaggerate depressive symptoms. Akiskal et al. [43] reported that depressed patients with high levels of neuroticism and introversion were more pathological than depressed patients without high levels of neuroticism and introversion. Therefore, behaviours related to introversion are obvious targets for psychological interventions and should also be focused on in outcome evaluation of medication treatment of depression. Increased introversion scores in remitted depressed patients may represent an increased risk of relapse.

We have previously attempted to identify “hidden” bipolarity in patients with unipolar depression by use of the Hypomania Checklist (HCL-32) [24]. In this study, 50% of the nonremitted unipolar patients had a “hidden” bipolar condition. This finding was in accordance with other studies [44]. As “hidden” bipolarity has been associated with poor treatment response to antidepressive medication [45], signs of bipolarity in depressed patients are an important clinical focus. In the present study, item No. 3 (Does your mood often go up and down?) from the Eysenck neuroticism scale was analysed separately. The results showed that 107 of the 239 patients (or 45%) gave a positive answer to this question and that these patients did have a significantly higher MES score (Table 7). Koukopoulus et al. [46] have in their review on mixed depressive states identified anxiety to be a core item within these bipolar states. Within the Hamilton Anxiety Scale (HAM-A_14_) we have shown that the following six items (HAM-A_6_) covering anxious mood, psychic tension, fears, difficulty in concentration, general muscular tension, and agitation are the core items [1]. Patients with a positive response to item No. 3 in the neuroticism scale (Table 7) score significantly higher on this subscale as well. 

The term “subthreshold bipolarity” covers both the inclination to be “moody” as well as risk-taking behaviour. In the component of active/elevated mood, subthreshold bipolarity is associated with extraversion as well as neuroticism. To make the correlation between the extraversion scale and the neuroticism scale as low as possible, Eysenck excluded the item of unstable mood as it relates to extraversion as well as neuroticism. A study by Barnett et al. [40] demonstrated that depression was associated with increased neuroticism and decreased extraversion, while manic symptoms were associated with increased extraversion and no reliable effect on neuroticism. Therefore, item No. 3 (Does your mood often go up and down?) may prove clinically relevant as a screening item when distinguishing unipolar depression from bipolar depression. The ability to distinguish between unipolar and bipolar depression using this single item that taps the interface of neuroticism and extraversion should be investigated in future studies adopting a longitudinal design in a clinical setting with depressed patients.

Although patients with previous bipolar affective disorder were excluded from the study, as evidenced by the studies above up to half of the sample may have had bipolar features nevertheless. Because “hidden” bipolarity is often associated with lack of treatment response in depression, and the 45% of the patients in this study that confirmed unstable mood experienced significantly higher depressive symptoms than the remaining 55% that disconfirmed unstable mood, item No. 3 (Does your mood often go up and down?) should be further investigated as screening for bipolarity in depression. 

## 5. Conclusion

In conclusion, this strict clinimetric study has shown the path from Eysenck's identification by principal component analysis of neuroticism and extraversion to their correct scientific measurement aspects within the item response theory model. The neuroticism scale contains an anxiety and a depression subscale. The extraversion scale should also be separated into an extraversion and an introversion subscale. In this clinimetric analysis we have illustrated that when items are phrased very similarly, the relationship might purely be a matter of semantics (high coefficient alpha) rather than of clinical validity. We have thus identified the item “Does your mood often go up and down?” as having a clear relationship to depression in our sample of patients outside the spectrum of schizophrenia

## Figures and Tables

**Table 1:** Eysenck Neuroticism N_23_ First principal component: Eigenvalue 6.83.

No.	Symptom	Mean score	Loadings
84 (D)	Are you sometimes bubbling over with energy and sometimes very sluggish?	0.83	0.31
47 (A)	Do you worry about your health?	0.68	0.39
88 (D)	Are you touchy about some things?	0.67	0.33
72 (A)	Do you worry too long after an embarrassing experience?	0.66	0.55
7 (D)	Do you ever feel “just miserable” for no reason?	0.64	0.64
34 (A)	Are you a worrier?	0.59	0.74
19 (A)	Are your feelings easily hurt?	0.53	0.55
41 (A)	Would you call yourself tense or “highly-strung”?	0.53	0.44
12 (D)	Do you often worry about things you should not have done or said?	0.53	0.59
80 (D)	Are you easily hurt when people find fault with you or the work you do?	0.52	0.45
58 (D)	Have you often felt listless and tired for no reason?	0.50	0.63
31 (A)	Would you call yourself a nervous person?	0.47	0.59
38 (A)	Do you worry about awful things that might happen?	0.49	0.53
3 (D)	Does your mood often go up and down?	0.45	0.71
23 (D)	Do you often feel “fed up”?	0.45	0.71
15 (A)	Are you an irritable person?	0.44	0.47
66 (D)	Do you worry a lot about your looks?	0.41	0.32
54 (A)	Do you suffer from sleeplessness?	0.36	0.39
75 (D)	Do you suffer from “nerves”?	0.36	0.59
68 (D)	Have you ever wished that you were dead?	0.33	0.48
62 (D)	Do you often feel life is very dull?	0.32	0.60
27 (D)	Are you often troubled about feelings of guilt?	0.31	0.67
77 (D)	Do you often feel lonely?	0.24	0.52

	Total score		

A: anxiety items.

D: depression items.

**Table 2:** Extraversion scale E_21_ First principal component: Eigenvalue 5.60.

No.	Symptom	Loadings
1 (R)	Do you have many different hobbies?	0.29
5 (E)	Are you a talkative person?	0.62
10 (E)	Are you rather lively?	0.68
14 (R)	Can you usually let yourself go and enjoy yourself at a lively party?	0.57
17 (E)	Do you enjoy meeting new people?	0.53
21 (I)	Do you tend to keep in the background on social occasions?	−0.65
25 (R)	Do you like going out a lot?	0.27
29 (I)	Do you prefer reading to meeting people?	−0.31
32 (E)	Do you have many friends?	0.42
36 (R)	Would you call yourself happy-go-lucky?	0.31
40 (R)	Do you usually take the initiative in making new friends?	0.27
42 (I)	Are you mostly quiet when you are with other people?	−0.68
45 (R)	Can you easily get some life into a rather dull party?	0.53
49 (R)	Do you like telling jokes and funny stories to your friends?	0.45
52 (E)	Do you like mixing with people?	0.64
56 (R)	Do you nearly always have a “ready answer” when people talk to you?	0.49
60 (E)	Do you like doing things in which you have to act quickly?	0.50
64 (R)	Do you often take on more activities than you have time for?	0.27
70 (E)	Can you get a party going?	0.67
82 (E)	Do you like plenty of bustle and excitement around you?	0.55
86 (E)	Do other people think of you as being very lively	0.65

E: the nine items in E_9_ (extraversion).

I: the three introversion items.

R: remaining extraversion items.

**Table 3:** Baseline characteristics of total number of patients.

Type of pain	Duration of pain (months) mean (s.d.)	Age (years) mean (s.d.)	Gender ratio females (%)
Headache (*n* = 114)	169.3 (152.4)	42.5 (13.1)	64
Burning mouth (*n* = 77)	53.7 (46.6)	61.9 (8.5)	92
Abdominal pain (*n* = 47)	79.3 (101.0)	48.1 (15.5)	70
Low back pain (*n* = 15)	31.1 (25.7)	43 8 (13.8)	60

**Table 4:** Eysenck Neuroticism Third principal component: Eigenvalue 1.32.

No.	Symptom	Negative loadings
54	Do you suffer from sleeplessness?	−0.41
68	Have you ever wished that you were dead?	−0.33
84	Are you sometimes bubbling over with energy and sometimes very sluggish?	−0.29
58	Have you often felt listless and tired for no reason?	−0.27
88	Are you touchy about some things?	−0.27
15	Are you an irritable person?	−0.26
7	Do you ever feel “just miserable” for no reason?	−0.21
62	Do you often feel life is very dull?	−0.14
3	Does your mood often go up and down?	−0.14
23	Do you often feel “fed up”?	−0.11

No.	Symptom	Positive loadings

47	Do you worry about your health?	0.49
66	Do you worry a lot about your looks?	0.33
72	Do you worry too long after an embarrassing experience?	0.32
77	Do you often feel lonely?	0.21
31	Would you call yourself a nervous person?	0.20
19	Are your feelings easily hurt?	0.19
38	Do you worry about awful things that might happen?	0.16
41	Would you call yourself tense or “highly-strung”?	0.15
75	Do you suffer from “nerves”?	0.11
80	Are you easily hurt when people find fault with you or the work you do?	0.08
34	Are you a worrier?	0.07
12	Do you often worry about things you should not have done or said?	0.07
27	Are you often troubled about feelings of guilt?	0.05

**(a):** 

	Coefficient of homogeneity	Cronbach's alpha
Extraversion scale E_21_	0.24	0.85
Extraversion subscale E_9_	0.39	0.80
Introversion I_3_	0.39	0.60

Coefficient of homogeneity.

0.30–0.39: just acceptable.

0.40–0.49: acceptable.

0.50 or higher: excellent.

Alpha: 0.80 or higher is excellent [1, 24].

**Table:** (b)

*N* = 234	Coefficient of homogeneity	Alpha coefficient
Neuroticism scale (N_23_)	0.34	0.75
N_9_ (anxiety)	0.32	0.83
N_14_ (depression)	0.38	0.88

**Table:** (a)

*N* = 234	MES	BDI_6_	HAM-A
Extraversion (E_9_)	−0.19	−0.23	−0.21
Introversion (I_3_)	0.29	0.31	0.31
*P*	<0.05	<0.05	<0.05

**Table:** (b)

*N* = 234	MES	BDI_6_	HAM-A
Neuroticism anxiety (N_9_)	0.43	0.55	0.47
Neuroticism depression (N_14_)	0.46	0.60	0.46

**Table 7:** The association between Item No. 3 of the Eysenck Neuroticism scale and the MES, HAM-A_14_, and HAM-A_6_.

Does your mood often go up and down?			
	MES mean (s.d.)	HAM-A_14_ mean (s.d.)	HAM-A_6_ mean (s.d.)
No			
*N* = 132	5.8 (4,1)	9.4 (5.2)	4.5 (5.2)
Yes			
*N* = 107	9.0 (4,3)*	13.5 (5.9)*	6.5 (3.1)*

*P < 0.001.
